# Decoding human cytomegalovirus for the development of innovative diagnostics to detect congenital infection

**DOI:** 10.1038/s41390-023-02957-9

**Published:** 2023-12-25

**Authors:** Shelley M. Lawrence, Tyler Goshia, Mridu Sinha, Stephanie I. Fraley, Marvin Williams

**Affiliations:** 1 University of Utah, College of Medicine, Department of Pediatrics, Division of Neonatology, Salt Lake City, UT USA; 2 Department of Bioengineering, University of California, San Diego, San Diego, CA USA; 3 MelioLabs, Inc., Santa Clara, CA USA; 4 University of Oklahoma, College of Medicine, Department of Obstetrics and Gynecology, Division of Fetal-Maternal Medicine, Oklahoma City, OK USA

## Abstract

**Abstract:**

Cytomegalovirus is the most common cause of congenital infectious disease and the leading nongenetic etiology of sensorineural hearing loss. Although most infected neonates are asymptomatic at birth, congenital cytomegalovirus infection is responsible for nearly 400 infant deaths annually in the United States and may lead to significant long-term neurodevelopmental impairments in survivors. The resulting financial and social burdens of congenital cytomegalovirus infection have led many medical centers to initiate targeted testing after birth, with a growing advocacy to advance universal newborn screening. While no cures or vaccines are currently available to eliminate or prevent cytomegalovirus infection, much has been learned over the last five years regarding disease pathophysiology and viral replication cycles that may enable the development of innovative diagnostics and therapeutics. This Review will detail our current understanding of congenital cytomegalovirus infection, while focusing our discussion on routine and emerging diagnostics for viral detection, quantification, and long-term prognostication.

**Impact:**

- This review highlights our current understanding of the fetal transmission of human cytomegalovirus.
- It details clinical signs and physical findings of congenital cytomegalovirus infection.
- This submission discusses currently available cytomegalovirus diagnostics and introduces emerging platforms that promise improved sensitivity, specificity, limit of detection, viral quantification, detection of genomic antiviral resistance, and infection staging (primary, latency, reactivation, reinfection).

## Introduction

Human cytomegalovirus (HCMV) is a common DNA herpesvirus that infects 60%-90% of adults worldwide, with a higher prevalence in persons of non-Caucasian backgrounds and lower socioeconomic status.^1,2^ HCMV transmission occurs mainly from persistent and prolonged viral shedding (months to years) by asymptomatic, seropositive individuals through contact with infected body fluids or organ donation.^3–5^ Conversely, congenital CMV (cCMV) results from viral transmission from the placenta to the fetus during pregnancy.^6^ While most cases of HCMV infection are mild or asymptomatic, deficiencies in host adaptive immune responses may allow an infection to progress to a life-threatening condition.

The natural history of HCMV infection has been defined into four subtypes: primary, latency, reactivation, and reinfection.^7–9^ *Primary* HCMV infection occurs when an individual acquires the virus for the first time and lacks previous immunity. The virus eventually enters a *latency* phase from which it can *reactivate* from stress or other medical conditions. The fourth subtype, *reinfection*, occurs following an infection with a new strain despite natural immunity (i.e., superinfection). These infection subtypes can complicate pregnancy, making HCMV the most common etiology of congenital infection.^10,11^

Viral shedding is estimated to occur in up to 40% of HCMV seropositive women and depends on their age group, socioeconomic status, education level, race, and ethnicity.^12^ In the United States, about 30,000 infants will be diagnosed with cCMV annually,^13^ with an associated cost of $2-$6.6 billion annually.^14^ Notably, more children are adversely affected by cCMV infection than more well-known conditions, including Down syndrome, fetal alcohol syndrome, and spina bifida.^15^ Although most neonates (90%) with cCMV demonstrate no clinical symptoms, about 8,000 infants will experience long-term neurodevelopmental impairments or may die from cCMV-associated complications.^13,16^

cCMV is also the leading nongenetic cause of sensorineural hearing loss (SNHL), occurring in almost half of symptomatic and 7% of asymptomatic cases.^16–18^ Notably, a quarter of neonates with cCMV who have normal hearing screen results after birth will develop SNHL later in childhood.^18^ While a primary maternal infection is generally associated with more severe sequelae in the offspring, prior maternal immunity is only partially protective and does not entirely shield the newborn from developing adverse neurologic conditions, including SNHL.^19^ Furthermore, cCMV with severe clinical features can occur irrespective of the trimester during which primary maternal infection occurs but is more common when the pregnant woman is infected during the first trimester.^20^

Like other *Herpesviridae* family members, individuals who acquire HCMV develop life-long infections due to its ability to establish latency in peripheral blood monocytes and CD34^+^ hematopoietic stem and progenitor cells (HSPCs).^21,22^ Infected myeloid cells are subjected to viral-induced modifications of host gene expression that cause cellular dysfunction and physiological changes that prolong the cell’s lifespan and allow the virus to disseminate undetected by immune surveillance mechanisms.^18,22^ Subsequent entry of infected monocytes into tissues causes their differentiation into either macrophages or dendritic cells, which drives viral reactivation and propagation.^23–25^

This Review will detail our current understanding of congenital cytomegalovirus infection, while focusing our discussion on routine and emerging diagnostics for viral detection, quantification, and long-term prognostication.

## Congenital cytomegalovirus infection

In developed countries, 4 in 10 women of reproductive age are seronegative for HCMV, and 5-10% of these women will experience a primary HCMV infection during their pregnancy.^26,27^ In the United States, seroprevalence increases with age and varies by race/ethnicity, resulting in the highest cCMV prevalence in black (9.5/1000 live births), followed by Hispanic white (3.0/1000 live births) and non-Hispanic white (2.7/1000 live births) newborns.^10^ These differences remain after controlling for household income level, education, marital status, area of residence, census region, family size, country of birth, and type of medical insurance.^2,28^ Although a heightened risk for in-utero HCMV transmission is observed in pregnancies complicated by maternal primary infection, nearly two-thirds of cCMV infections occur in seropositive women.^9^ Fetal infection in these cases results from reinfection or reactivation of latent virus.^9^ Despite the high rate of cCMV observed in mothers with pre-existing maternal immunity, fetal transmission is low (0.1%-1%),^29^ and the prevalence of congenital infection is 0.4-6.1%.^30^

The gold standard for diagnosing fetal HCMV infection is the detection of viral DNA in the amniotic fluid via amniocentesis after the 21^st^ week of gestation and at least six weeks after a maternal lytic infection.^31^ Mechanisms by which HCMV is transmitted to the fetus remain poorly understood, but chronic villitis is commonly observed upon placental histology.^32,33^ Investigations have been limited to human explant models and placental primary cell line cultures.^34–36^ Despite these limitations, the role of trophoblasts is particularly interesting as these cells are the first embryonic structure to differentiate after fertilization (around day four) and are necessary for uterine implantation.

**Fig. 1:**
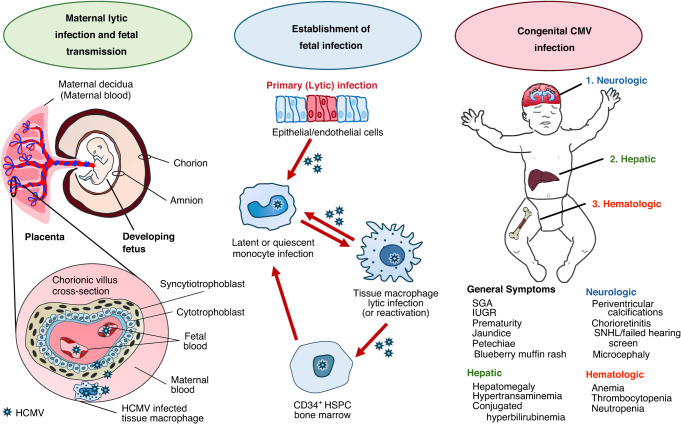
Pathophysiology of congenital cytomegalovirus infection. (Right, Maternal Infection) Maternal lytic infection is caused by primary infection, reactivation of latent infection, or reinfection with a new strain (i.e., superinfection). The inner cytotrophoblast is surrounded by placental fibroblasts and macrophages that directly contact the developing fetal blood vessels, while the outer syncytiotrophoblast layer is thought to be human cytomegalovirus (CMV) permissive.^37,38^ Once in the fetal bloodstream, the virus can spread to susceptible cell types, including fetal endothelial cells, fibroblasts, and smooth muscle cells, mainly through cell-to-cell transmission but also by cell-free mechanisms.^46,47^ (Middle, Fetal Infection) HCMV develops life-long infection due to its ability to establish latency in peripheral blood monocytes and CD34^+^ hematopoietic stem and progenitor cells (HSPCs).^21,22^ Subsequent entry of infected monocytes into tissues causes their differentiation into either macrophages or dendritic cells, which drives viral reactivation and propagation.^23–25^ (Left, Neonatal Findings) Clinical signs of congenital cytomegalovirus (cCMV) infection included small for gestational age (SGA), intrauterine growth restriction (IUGR), preterm birth, microcephaly, congenital hydrocephalus, chorioretinitis, thrombocytopenia, anemia, hypertransaminemia, hepatomegaly, and sensorineural hearing loss (SNHL).^9,51^

In humans, the trophoblast proliferates and differentiates into two cell layers approximately one week after fertilization: (a) the outer syncytiotrophoblast (ST), containing multinucleated, mitotically inactive fetal cells that are in direct contact with maternal blood, creating a physical barrier within the villous placenta; and (b) the inner cytotrophoblast (CT) that originates during the first and second trimesters and consists of a single layer of immature, mitotically active, mononuclear cells^37^ (Fig. 1). The CT is surrounded by placental fibroblasts and macrophages that directly contact the developing fetal blood vessels.^37,38^ Maturation of the ST during pregnancy may contribute to gestational age-related differences in fetal CMV transmission,^38^ as infection of the ST occurs at a higher frequency and progresses more rapidly during the early stages of pregnancy. However, the ability of these highly permissive cells to establish a lytic infection of the surrounding fibroblasts is impaired due to the presence of suboptimal viral titers and low levels of maternal neutralizing antibodies.^38^ Differential expression of platelet-derived growth factor receptor-α (PDGFR-α) by extravillous (low) versus villous (high levels) trophoblasts may also affect the ability of cytomegalovirus to gain entry into these cells and establish viral replication.^39^

Infection of placental pericytes, located abluminal to microvascular endothelial cells, may also be a source for HCMV lytic infection during maternal viremia.^40,41^ Together with the trophoblast, placental pericytes contribute to the blood-placental barrier and are essential for placental endothelial cell proliferation,^42^ vascular development and angiogenesis,^42^ and microvascular integrity and stability.^43,44^ Aronoff and colleagues^36^ showed in human tissue explants and primary placental cell-line cultures that HCMV infection of pericytes causes cell loss at proximal sites of the placental vasculature, leading to regional inflammation, impaired tissue perfusion, enhanced angiogenesis, and microcirculatory abnormalities. HCMV may also replicate in the maternal decidua, generating a viral reservoir that can increase fetal transmission risks during primary infections.^45^

HCMV is transmitted to the developing fetus by hematogenous dissemination via the placenta during maternal viremia. Once in the fetal bloodstream, the virus can spread to susceptible cell types, including fetal endothelial cells, fibroblasts, and smooth muscle cells, mainly through cell-to-cell transmission but also by cell-free mechanisms.^46,47^ While fibroblasts and smooth muscle cells enable robust viral replication, mucosal epithelial and endothelial cells experience only low-level virus shedding.^48,49^ As a result, a weaker host immune response is elicited that supports the establishment of chronic infection, prolonged viral shedding, and HCMV transmission to other susceptible hosts.^48^ This phenomenon is demonstrated clinically by infectious viruria of infants with cCMV and viral shedding in the breast milk of seropositive lactating women.^50^

Clinical findings of cCMV can vary greatly, from an asymptomatic infection to a life-threatening illness. Scaramuzzino and colleagues^9^ evaluated signs and symptoms present at birth in 53 symptomatic neonates divided by the type of maternal infection [i.e., primary (n = 40) versus secondary (n = 13) infection]. These investigators found that only the incidence of unilateral SNHL differed between the two groups (17.5% in symptomatic versus 46.2% in asymptomatic cCMV cases, p = 0.037). Other findings included small for gestational age, preterm birth, microcephaly, congenital hydrocephalus, chorioretinitis, thrombocytopenia, anemia, hypertransaminemia, hepatomegaly, SNHL, neurologic signs (i.e., hypotonia, hypertonia, and seizures), and abnormal findings on cranial ultrasound or MRI (Fig. 1).^9,51^

## Neonatal screening for cCMV

The American Academy of Pediatrics and Joint Committee on Infant Hearing released a Position Statement on “*The Principles and Guidelines for Early Hearing Detection and Intervention Programs”* in 2007. While this document does not provide specific recommendations for cCMV screening, it does advocate for at least one diagnostic audiology assessment by 24 to 30 months of age in all infants, with “earlier and more frequent assessments in those diagnosed with cCMV”.^52^ Conversely, consensus recommendations that specifically address cCMV screening in pregnant women and their offspring were subsequently released by the International Congenital Cytomegalovirus Recommendations Group (2017)^53^ and the Academy of Audiology (2023).^54^ Screening recommendations from these groups are summarized in Table 1. Notably, universal newborn cCMV screening is not currently recommended.^53,54^

**Table 1:** Current Screening Recommendations for Fetal and Neonatal Congenital Cytomegalovirus.^53,54^

Indications	Diagnostic Test	Result Interpretation
**Maternal**		
1. Flu-like symptoms during pregnancy (i.e., fever, fatigue, and headache) not attributable to another infection 2. Ultrasound (or fetal MRI) are suggestive of fetal CMV infection	CMV-specific IgG, IgM, and IgG avidity and/or	1. For previously known CMV-seronegative pregnant women, a primary infection is diagnosed via detection of CMV-specific IgM in serum 2. If CMV status is unknown, then primary maternal infection is indicated by the detection of CMV-specific IgM and IgG and low-to-moderate avidity
	Real-time PCR detection of CMV in the amniotic fluid	Fetal CMV infection can be made if the PCR detects CMV after 20-21 weeks of gestation and at least 6 weeks from the time of maternal infection
**Neonatal**		
1. Maternal diagnosis of CMV infection during pregnancy 2. Clinical signs and symptoms, including failure to pass the newborn hearing screen on two or more attempts (one or both ears), SGA, IUGR, thrombocytopenia, hyper-transaminemia, hepatomegaly, characteristic petechial rash, microcephaly or macrocephaly, and/or abnormal findings on cranial ultrasound or MRI	Real-time PCR of saliva, urine, or both	A positive PCR assay indicates cCMV when obtained within the first 3 weeks of life

*PCR* polymerase chain reaction, *CMV* cytomegalovirus, *cCMV* congenital cytomegalovirus, *SGA* small for gestational age, *IUGR* intrauterine growth retardation, *MRI* Magnetic Resonance Imaging.

## Diagnostics

Detecting cytomegalovirus in culture or HCMV DNA in the blood, urine, saliva, or cerebrospinal fluid (CSF) within the first three weeks of life is necessary to diagnose a congenital infection.^55^ After this time, differentiation between a congenital versus postnatally acquired infection is difficult. Technologies used to identify HCMV have evolved over the last half-century, and nucleic acid amplification-based tests [i.e., polymerase chain reaction (PCR)]^56^ have become the current standard for routine sample testing in multiple tissue matrices.^57–59^ Technologies that incorporate NAAT-based approaches can be qualitative (detection of CMV only or yes/no answer) or quantitative (CMV detection with calculation of viral load). Quantitative approaches can be used to determine a patient’s response to antiviral therapy, the likelihood that the viral load correlates with the clinical presentation, the appropriate time to discontinue treatment, and the risks for possible relapse of infection.^60^

Emerging diagnostics promise the ability to not only rapidly identify HCMV DNA from multiple tissue matrices but also provide information regarding the cytomegalovirus strain(s) and antiviral resistance pattern(s) through ultrasensitive detection of single nucleotide-level modifications of the viral genome that are simultaneously analyzed by artificial intelligence algorithms. Innovative platforms also have the potential to stage the infection (primary, latency, reactivation, or reinfection) by scrutinizing host and viral gene expression signatures and quantifying viral load. Systemic reviews and metanalysis for cCMV diagnostics are lacking, with a single peer-reviewed publication from Wang and colleagues^61^ regarding the performance of PCR on dried blood spot (DBS). These authors included 15 studies, with total of 26,007 neonates, and calculated a pooled sensitivity and specificity of 0.844 (95% CI = 0.812-0.872) and 0.999 (95% CI = 0.998-0.999), respectively. Below is a summary of current diagnostics, organized into (a) prenatal detection, (b) diagnosis of neonatal cCMV, and (c) predictors and/or biomarkers of severity of disease, antiviral resistance, and outcome. Emerging technology with promising preliminary results for prenatal detection and diagnosis of cCMV is discussed separately. An overview of current and emerging diagnostics is provided in Table 2.

**Table 2:** Overview of past, present, and emerging cytomegalovirus diagnostics.

Method	Availability	Principle	Sample Processing	Turnaround Time	Advantages	Disadvantages
**Microscopy & Histopathology**	Commercial and University Laboratories	Detection of HCMV-infected cells	Tissue staining with microscopic review	24 h - 48 h	Enables determination of HCMV-mediated end-organ disease	Tissue samples usually obtained by invasive methods; skilled professionals for analysis
**Viral Culture**	Commercial and University Laboratories	Growth of virus in cell culture	Cell culture with microscopic review	Up to 28 days	Can isolate HCMV-specific strain for phenotypic analysis of antiviral resistance	More labor intensive than NAAT assays; long turnaround time may inhibit clinical utility
**Serologies**	Commercial and University Laboratories	Detection of host antibodies against HCMV-specific antigens (IgG/IgM)	Patient serum samples	6 h	May be helpful to determine stage of infection	Not useful for cCMV; interpretation in immunocompromised hosts may be problematic
**Nucleic Acid Amplification Test**	Commercial and University Laboratories	Detection of HCMV DNA	Can be used to analyze various tissue matrices	1 h - 4 h	Quantitative approaches can be reported as CMV copies/mL; can provide antiviral genomic information; rapid; easy to use assays; highly sensitive	Differences in assay types may limit comparisons between laboratories; low viral loads may result in false-negative results
**High Resolution Melt**	Research Based	Detection of HCMV DNA at the single nucleotide level	Can be used to analyze various tissue matrices	4 h	Quantitative; can provide antiviral genomic information; rapid; easy to use assays; highly sensitive; can be multiplexed for broad pathogen detection	Studies on cCMV and other herpesviruses are ongoing
**Next Generation Sequencing**	Primarily University and Research Laboratories	Sequencing of the host genome and microbiome, including viral genome	Can be used to analyze various tissue matrices	24 h - 5 days	Quantitative; can provide antiviral genomic information; highly sensitive; can be multiplexed for broad pathogen detection	Expensive; time intensive; requires highly trained individuals and equipment that will limit dissemination to lower resource settings
**POC Biosensors**	Research Based	Detection of HCMV via reactions with viral components	Can be used to analyze various tissue matrices	<1 h	Very sensitivity, short turnaround time, no requirement of laboratory settings, and inexpensive	Studies on cCMV and other herpesviruses are ongoing
**CRISPR-Based**	Research Based for cCMV	Detection of HCMV DNA	Can be used to analyze various tissue matrices	<1 h	Very sensitivity, short turnaround time, no requirement of laboratory settings, and inexpensive	Studies on cCMV and other herpesviruses are ongoing

*HCMV* human cytomegalovirus, *cCMV* congenital cytomegalovirus, *NAAT* Nucleic acid amplification test. *POC* point of care, *CRISPR* clustered regularly interspaced short palindromic repeats.

## Prenatal detection

### Serologies

Serologies that detect HCMV antibodies (IgM and IgG) via enzyme-linked immunosorbent assay (ELISA) are widely available from commercial and university laboratories. IgG avidity tests, which measure the binding strength between IgG antibodies and the virus, are also routinely offered and may be used to help distinguish between a primary and past infection.^62^ That is, low binding strength (low avidity) is usually observed in a primary infection, while high avidity is associated with later stages of infection. However, serologies are not routinely used to diagnose cCMV, as IgG does not discriminate between a maternal or neonatal infection, and IgM is not sufficiently sensitive or specific for congenital infection.^62,63^

Identification of antibodies against CMV-specific antigens has also been evaluated clinically because of their ability to differentiate between primary and established HCMV infections. Muller and colleagues^64^ reported on the use of anti-p52 IgM and anti-gB IgG compared to routine IgG, IgM, and avidity assays during pregnancy to return “conclusive results” (i.e., susceptible to infection, acute primary infection, recurrent infection/reactivation, or past infection). These investigators found the use of anti-p52 IgM and anti-gB IgG produced conclusive results in 513/553 [92.8%, 95% confidence interval (CI): 90.3-94.7%] compared to routine IgG, IgM, and avidity testing 468/553 (84.6%, 95% CI: 81.4%-87.4%). In this study, only 417/553 (75.4%, 95%CI: 71.7%-78.8%) samples were classified into the same diagnostic category using both approaches.

### NAAT-based testing

In HCMV-infected patients, viremia involves the intracellular infection of myeloid cells and the small, fragmented, non-infectious viral DNA found in the plasma. Differences between the two should be considered when ordering routine NAAT assays, as their results may have significant clinical implications.^60^ HCMV DNA loads in plasma are usually less than that reported in whole blood, as the size of the amplicon used in the assay can vary considerably (up to 100-fold in copy number between assays, or from 50 to 350 bp).^60,65–67^ This concept was demonstrated by Peddu and colleagues,^65^ who investigated the use of cfDNA for maternal HCMV screening. cfDNA, which is routinely sent for non-invasive prenatal aneuploidy screening, was shown to be a viable sample source with high sensitivity when a small amplicon [≤ 86 base pairs (bp) compared with standard PCR amplicons > 105 bp] was used. These investigators showed that the HCMV-associated cfDNA fragment size was substantially shorter than human-specific sources (103 vs. 172 bp, p < 0.0001).

## Diagnosis of neonatal cCMV

### Viral cultures and shell vial assays

Viral cultures, employing fibroblast monolayers, are also used in clinical laboratories. However, viral cultures lack sensitivity and must be maintained for more than 28 days before being considered negative.^68^ Consequently, the shell vial assay gained popularity in the 1990s and 2000s as the primary test for cCMV because it was a more rapid and sensitive method than culture-based techniques, with results reported in under 48 hours.^69^ This test utilized low-speed centrifugation of newborn urine samples on a fibroblast monolayer and incorporated specific monoclonal antibodies to detect viral antigens produced during the early stages of infection coupled with a second antibody labeled either with a fluorescent dye or an enzyme. The viral load could then be estimated by microscopically counting the number of labeled cells.^69^ However, nucleic acid amplification-based technologies replaced shell vial assays over a decade ago, as these assays offered improved sensitivity and ease of use.

### NAAT-based testing and cCMV

Food and Drug Administration (FDA)-approved commercially available NAAT kits are listed in Table 3. Only two are commercially available for the diagnosis of cCMV: Alethia® by Meridian Bioscience, Inc. and Simplexa^™^ Congenital CMV Direct by DiaSorin Molecular. While both assays can be used to test saliva swab samples, only Simplexa^™^ is also approved to test urine samples. Neither are cleared for whole blood, plasma, or dried blood spot (DBS) card testing. These two assays differ in the technique used to amplify the target DNA. Alethia® employs a method known as loop-mediated isothermal amplification (LAMP), which can amplify DNA up to a billion copies in less than an hour, compared to a million copies typically generated by PCR.^70^ LAMP also utilizes several primers (from four to six) compared to only two in most PCR kits and can be performed with basic laboratory equipment (i.e., dry block heater or water bath) instead of advanced heating equipment.^70^ However, a recent study by Atwood and colleagues^71^ found that the Alethia assay had a false-positive rate of 4.5% to 6.2% (n = 696 saliva specimens), which was higher than the 0.2% reported in FDA claims. Therefore, laboratories that use this assay should consider prospective quality management to evaluate all positive results.^71^

**Table 3:** FDA-approved nucleic acid amplification-based test for HCMV.

Product & Company	Patient Population and Product Information	Assay Technique and Output	Input
Abbott RealTime CMV **(Abbott Molecular, Inc.)**	Hematopoietic stem cell transplant patients https://www.molecular.abbott/us/en/products/infectious-disease/realtime-cmv	qPCR Quantitative to assess response to therapy	EDTA plasma
Alethia® **(Meridian Bioscience, Inc.)**	Neonates less than 21 days of age https://www.meridianbioscience.com/diagnostics/disease-areas/pediatric-neonatal/cmv/?country=US	LAMP Qualitative	Saliva swab
Artus **(Qiagen)**	Solid organ transplant patients https://www.qiagen.com/us/products/diagnostics-and-clinical-research/transplant/artus-viral-load/artus-cmv-rgq-mdx-kit-us	qPCR Quantitative to assess response to therapy	EDTA plasma
Alinity M CMV **(Abbott Molecular, Inc.)**	Hematopoietic stem cell transplant and Solid organ transplant patients https://www.molecular.abbott/int/en/Alinity-m-CMV-Assay	qPCR Quantitative to assess response to therapy	EDTA plasma
Aptima CMV Quant Assay **(Hologic, Inc.)**	Hematopoietic stem cell transplant and Solid organ transplant patients https://www.hologic.com/hologic-products/molecular-diagnostics/aptima-cmv-quant-assay	qPCR Quantitative to assess response to therapy	EDTA plasma
COBAS AmpliPrep **(Roche Molecular Systems)**	Solid organ transplant patients https://diagnostics.roche.com/us/en/products/params/cobas-ampliprep-cobas-taqman-cmv-test.html	qPCR Quantitative to assess response to therapy	EDTA plasma
COBAS CMV Test **(Roche Molecular Systems)**	Hematopoietic stem cell transplant and Solid organ transplant patients https://diagnostics.roche.com/us/en/products/params/cobas-cmv.html	qPCR Quantitative to assess response to therapy	EDTA plasma
NucliSens CMV pp67 **(Organon Teknika Corp.)**	Adult transplant donors and HIV-infected patients for active (acute or reactivated) infection ^a^No website available	NASBA Qualitative	EDTA whole blood
Simplexa^™^ Congenital CMV Direct **(DiaSorin Molecular LLC)**	Neonates less than 21 days of age https://us.diasorin.com/en/molecular-diagnostics/kits-reagents/simplexa-congenital-cmv-direct-kit	Real-time PCR Qualitative	Saliva swabs and urine

*LAMP* loop-mediated isothermal amplification, *qPCR* quantitative polymerase chain reaction, *HIV* human immunodeficiency virus,

*NASBA* Nucleic acid sequence-based amplification, *EDTA* Ethylenediaminetetraacetic acid.

^a^See reference by Witt and colleagues.^110^

Although the FDA-cleared DiaSorin PCR assay reports qualitative results for detecting CMV, it also employs a quantitative technique inherent to real-time PCR [i.e., measuring cycle threshold (Ct) values]. These values, along with the amplification curves, are visible within the assay’s software. This feature gives laboratories the necessary data to assess a sample’s viral load. A recent study highlighted the assay’s versatility by demonstrating 100% sensitivity and specificity when the Ct cutoff was adjusted from the FDA-approved ≤37.5 to an Investigational Use Only assay definition of ≤42.0 because it enabled the detection of very low HCMV loads.^72^

Many large clinical laboratories utilize their own NAAT assays that are developed and validated in-house.^73^ As a result, individual assays may differ regarding how the nucleic acid is extracted, primer selection for amplification, test chemistry, and instrumentation.^60^ In November 2010, the World Health Organization approved an international standard for CMV NAAT-based assays. This standard allows individual laboratories and manufacturers to assess the accuracy of their viral load calculations and to calibrate their assays to the new standard [reported as international units (IU)/mL rather than copies/mL].^74^

Infants with cCMV persistently shed very high levels of HCMV in their saliva and urine (median >12 months).^75^ Saliva is collected by oral (not throat) swab and is usually more convenient to obtain than urine in the first 24 hours of life, as urine production is limited. False-positive saliva PCR results are possible in breastfeeding infants if a seropositive mother is shedding the virus, so confirmatory testing by urine PCR is recommended. Notwithstanding, PCR testing of neonatal saliva is reported to have high sensitivity (92.9%-97%) and specificity (99.9%) as a screening method and has been validated in large population-based cohort studies.^58,76^ Urine has a sensitivity of 98.8%-100% and specificity of 95%-99.1%.^56,77^ However, HCMV levels in the blood are much lower than in saliva or urine, which may contribute to a lower sensitivity of PCR testing of DBS cards collected at birth.^6,55,58,78^

Digital PCR (dPCR) maybe a more precise, reproducible, and quantifiable assay than real-time quantitative PCR (qPCR), especially in samples with low viral load. Unlike qPCR, which employs a single well to analyze nucleic acid samples, dPCR partitions the sample into tens of thousands of individual reactions to improve the sensitivity and quantification of the assay.^79^ Nonetheless, a study by Yamaguchi and colleagues^80^ showed no difference in blood CMV DNA loads measured by dPCR and qPCR, with or without clinical findings in 39 neonates with cCMV (21 symptomatic and 18 asymptomatic). However, developmental delay at 36 months was more accurately predicted by dPCR results, especially in patients with high HCMV DNA loads (≥2950 copies/mL).

Therefore, when utilizing NAAT, clinicians should consider the following when making clinical management decisions the: (1) type of specimen (i.e., urine, saliva, whole blood, or plasma), (2) limits of detection and quantification chosen by the clinical laboratory, (3) linear range of the assay, and (4) reproducibility within the institution.^57,59,81,82^ While whole blood allows for the determination of both the cell-free and cell-associated HCMV DNA, serum or plasma is used to detect only the cell-free fraction.

## Predictors and/or biomarkers of severity of disease, antiviral resistance, and outcome

### Microscopy and histopathology

CMV derived its name “cytomegalovirus” from changes in the host cell following viral infection. Specifically, CMV induces the development of one or more intracytoplasmic inclusion bodies, consisting of newly produced viruses and lysosomes, and a sizable intranuclear inclusion body that together causes the infected cell to become voluminous or attain cellular cytomegaly.^83^ HCMV can be identified by hematoxylin and eosin (H&E) staining of tissues, resulting in the pathognomonic appearance of cytomegalic cells with ‘owl eye’ inclusions. Detection of HCMV-infected cells in various tissues is the standard for diagnosing end-organ disease, but is associated with low sensitivity and requires trained personnel to analyze the tissue microscopically.^69^ In neonates, end-organ disease is generally correlated with clinical findings in association with hepatic and renal function tests, cranial ultrasound with or without magnetic resonance imaging, and complete blood counts.

### Inflammatory cytokines, proteomics, and gene expression panels

Investigations of host inflammatory biomarkers (i.e., cytokines, chemokines) to distinguish between symptomatic and asymptomatic cCMV cases have shown no discernable differences in immune responses.^18^ However, increased interferon (IFN) levels, a critical human innate immune response to viral infection, have been reported in cCMV-infected fetuses during pregnancy.^7^ IFN is essential for the repression of viral transcription, with HCMV reactivation associated with inhibition of IFN signaling.^22^

Proteomic analysis of the amniotic fluid of fetuses diagnosed with severe cCMV infection has also been investigated. Vorontsov and colleagues^84^ reported on the production of two proteins that are highly predictive of cCMV severity, retinoic acid receptor 2 (chemerin) and galectin-3-binding protein (Gal-3BP). An analysis of an independent validation cohort to differentiate between seventeen fetuses with severe cCMV from 26 fetuses with asymptomatic cCMV showed the combination of these two proteins had a sensitivity of 88.2%, specificity of 96.2%-100%, positive predictive value of 93.8%-100%, and negative predictive value of 92%.

Alternatively, Ouellette and colleagues^18^ investigated gene expression profiling in a preliminary study involving eighty neonates with cCMV (49 symptomatic, 31 asymptomatic). These investigators identified a 16-gene classifier signature (i.e., CD40, MYST2, LOC286135, JMJD2A, RABGAP1, RAB9B, AK3L1, MATR3, ARHGEF9, C10orf59, LOC645431, MPDU1, PAXIP1, CLEC4G, GLCCI1, and LEO1) through random forest analysis of samples from symptomatic and asymptomatic cCMV-infected neonates that predicted the development of SNHL with an accuracy of 92%. In this study, transcripts related to IFN were overexpressed in the top ten genes. Similar to previous studies, the 16-gene classifier could not distinguish between asymptomatic and symptomatic cases, supporting the premise that cCMV is a spectrum of clinical disease as opposed to discrete entities.

### NAAT and the detection of antiviral resistance in neonates with cCMV

While routine monitoring of viral burden is not currently advocated for neonates with moderate to severe symptomatic cCMV disease treated with ganciclovir (GCV) or valganciclovir (VGCV) for less than six months, recent investigations have reported antiviral resistance with prolonged courses (>6 months). Torii and colleagues^85^ employed long-read DNA sequencing of *UL97* and *UL54* in the blood samples of eleven patients. One patient with increasing viral load was found to have two drug-resistant mutations (*UL54V823A* and *UL97A594V*). This patient’s viral burden subsequently subsided with the cessation of GCV/VGCV. Likewise, Garofoli and colleagues^86^ reported two of nine severe cCMV cases that developed VGCV resistance associated with *UL97* gene mutations, leading these authors to advocate for viral load and antiviral resistance monitoring (alongside SNHL and neurodevelopmental improvements), especially when prolonged courses are  utilized.

## Emerging technology with promise for prenatal CMV detection and diagnosis of cCMV

### High-resolution melt analysis

High-resolution melt (HRM) analysis offers a promising high-throughput methodology for cytomegalovirus screening. The BioFire FilmArray® ME panel is FDA-approved to detect HCMV and 14 other organisms by relying on the unique melting temperatures of each organism-specific amplicon resulting from targeted amplification. Its clinical performance has not been studied extensively yet, but early reports suggest promising performance of 100% (95% CI: 43.9%-100%) sensitivity and 99.8% (95% CI: 99.4%-99.9%) specificity.^87–89^ Bispo and colleagues^90^ have also developed a highly sensitive multiplex HRM platform capable of simultaneously detecting and discriminating HCMV, HSV-1, HSV-2, varicella-zoster virus (VZV), and *Toxoplasma gondii* from the aqueous humor, undiluted vitreous, and diluted vitreous washings. This assay was highly sensitive, with a limit of detection of 20 genome copies for herpesviruses and 200 genome copies for *T. gondii*. However, this study underscores a significant aspect of HCMV diagnostics, namely the importance of employing a comprehensive detection and discrimination approach to identify other probable pathogens due to confounding and overlapping features from clinical findings alone.

The main limitations often addressed for HRM are the lack of breadth of the assays or issues with deconvoluting melt curves that could result from the amplification of two different targets in the same sample. Advancing broad-based HRM to a digital platform where individual genomes are compartmentalized into separate massively parallelized reactions should overcome these issues.^91^ For this reason, next-generation HRM technology that combines melt analysis with digital (d)PCR is emerging and may increase cost-effectiveness as a function of its lower complexity.^91–95^ Not only does utilizing digital HRM (dHRM) allow for increased sensitivity compared to qPCR HRM, but one of its main features is absolute quantification, which may hold significant value for outcome prediction and treatment stratification in cCMV. Despite the lack of published studies regarding the clinical performance of dHRM to diagnose cCMV, dHRM possesses desirable rapid, sensitive, and broad-profiling attributes for a cCMV diagnostic. Current investigations are ongoing in this regard.

### Next-generation sequencing

Next-generation sequencing (NGS) enables early broad-based qualitative and/or quantitative description of the host’s microbiome in a single test, including comprehensive profiling of the viral genome. Quantitative analysis depends on the depth of sequence data collected and the quality of the exposed target.^96^ It also facilitates the detection of genetic variants and antiviral resistance mutations. The use of NGS to identify viral diversity and its association with symptomatic cCMV infections and SNHL was recently reported by Dobbins and colleagues.^97^ These investigators analyzed CMV DNA from neonatal urine specimens (17 asymptomatic and 13 symptomatic cases) with 93% coverage of the CMV genome. They found CMV genes *UL48, UL88, US19*, and *US22* had a greater nucleotide diversity in symptomatic infants, while *UL57, UL20, UL104, US14, UL115*, and *UL35* had increased diversity in infants with SNHL.

NGS has also demonstrated superiority to Sanger sequencing in identifying antiviral drug resistance genes in immunocompromised adult and pediatric patients with HCMV infections.^98^ Nevertheless, Sanger sequencing remains the current gold standard for genotypic detection of cytomegalovirus antiviral resistance and functions by identifying mutations in PCR-amplified UL97 and UL54 gene segments.^99^ However, this approach has low sensitivity when viral loads <1000 HCMV copies/mL and fails to detect mutations in up to 20% of the viral population.^99^

Metagenomic NGS (mNGS) has also been investigated as a potential diagnostic for cCMV. In a prospective study by Ge and colleagues,^100^ mNGS was performed simultaneously with conventional tests (i.e., biochemical tests, culture, smear, and HSV-PCR) to identify central nervous system (CNS) infections in patients admitted to the neonatal intensive care unit (NICU). Whole exome sequencing (WES) was also completed on corresponding blood samples for patients with confirmed CNS infection and those with an unclear diagnosis. Eighty-eight patients were enrolled in this eight-month study, and 101 CSF samples were analyzed. Results showed that the diagnostic yield of mNGS was 19.8% (20/101) compared to 4.95% (5/101) for conventional methods. Five patients were etiologically identified by WES alone, and both mNGS and WES diagnosed one patient. However, mNGS identified three cases of cytomegalovirus that were not detected by conventional methods, demonstrating the potential for this technology to be a sensitive and highly specific test for cytomegalovirus detection among other potential pathogens.

Rapid whole genome sequencing (rWGS) has also been used to analyze blood samples from critically ill pediatric patients (1 day to 18 years) for DNA consistent with CMV infection by Ramchandar and colleagues.^101^ Of 669 patients who completed rWGS, 28 patients (4.2%) were CMV-positive, with a high correlation (R^2^ > 0.99, p < 0.001) to the corresponding CMV-qPCR DNA assay. Six of the 28 patients had clinical characteristics suggestive of symptomatic CMV infection, but only three were evaluated for CMV infection by PCR. A single patient was started on antiviral treatment prior to obtaining the rWGS results.

NGS technologies still face challenges for integration into regular patient care, such as limited testing access, technical expertise, exorbitant expense, and lengthy turnaround times. To date, NGS has yet to establish itself as robust enough to reliably define a true positive result without the requirement of confirmatory testing. There is also a need for more applied research explicitly conducted in the neonatal population. Consequently, additional research is necessary to assess the practical implications of NGS testing in the neonatal diagnostic setting and to determine the most effective strategies for its utilization in conjunction with conventional microbiological methods.

### Point-of-Care (POC) devices

#### Biosensors

A biosensor is a device that measures biological or chemical reactions by generating a signal proportional to the concentration of an analyte in the reaction.^102^ Commonly used point-of-care (POC) biosensors in the NICU are glucose and i-STAT blood analyzers.^103^ Biosensors for cCMV aim to address significant concerns of a rapid turnaround time and lack of adequate sensitivity, specificity, and cost-effectiveness. Many different analytical methods have been employed for HCMV biosensors, including label-free electrochemical, optical, piezoelectric, and immunosensors.^102^

Among biosensor methods, electrochemical approaches are leading the way in providing selective, sensitive, and rapid diagnosis of HCMV. Researchers have developed biosensors that eliminate the need for nucleic acid amplification or surface modification, making the diagnostic process faster, simpler, and more cost-effective. One method Chang and colleagues^104^ developed uses a surface plasmon resonance (SPR) biosensor, which detects changes in the refractive index caused by binding events between analytes and receptors on a metallic sensor surface. Their biosensing platform detects CMV-specific microRNAs (UL22A-5P and UL112-3p) with a coefficient of determination, *R*^2^, equal to 0.9961 for UL22A-5p and 0.9985 for UL112-3p. The limit of detection (LOD) was calculated to be 108 femtometers (fM, or 10^-15^ meters) for UL22A-5p and 24fM for UL112-3p. A proof-of-concept study involving four neonates (two cCMV-positive and two negative) demonstrated its ability to distinguish between healthy and HCMV-infected newborns within one hour.^104^

Alternatively, paper-based biosensors have extraordinary potential for widespread implementation because they are disposable, cost-effective, rapid, user-friendly, and have minimal volume sample requirements.^105^ One immunosensor created by Alba-Patiño and colleagues^105^ tests for HCMV in plasma samples by incorporating a detection reservoir for glycoprotein B, a CMV-specific glycoprotein that is vital for its entry into host cells. This platform was shown to have a LOD of 0.03 ng/mL and a total assay time of 12 minutes in mock HCMV-spiked blood samples. This test was also not affected by the long-term storage of the reservoirs without preservatives at room temperature. However, the performance of this biosensor in human clinical trials remains pending.

#### Clustered regularly interspaced short palindromic repeats (CRISPR)-based technologies

Recently, the CRISPR diagnostic field has rapidly expanded, leading to several FDA-authorized SARS-CoV-2 (COVID-19) tests and the development of other viral platforms. CRISPR-based viral diagnostic approaches have been combined with nucleic amplification-free electrochemical, paper-based biosensing, or nucleic acid amplification technologies (including isothermal amplification), which eliminates the requirement for laboratory heat cyclers - a major cost and ease-of-use impediment for widespread clinical implementation.^106^ CRISPR technology has also been shown to be highly adaptable and scalable, simultaneously detecting multiple pathogens or genetic variants within a single clinical sample and being easily reprogrammable to target different strains or variants as they emerge. The main benefits of CRISPR diagnostic tools for neonatal health are their ultra-sensitivity, short sample-to-answer time, no requirement of laboratory settings, and promise of being more affordable than conventional methods. Monk and colleagues^106^ evaluated using a CRISPR-Cas12a CMV rapid diagnostic (turnaround time of 90 minutes) on CMV-spiked human saliva and urine samples using UL123 and US28 target sites. Detection in urine was more robust than in saliva, with limits of detection ranging from 0.1 to 100 IU/mL depending on CMV strain and sample type. As such, several active CRISPR diagnostic clinical trials for detecting HCMV may soon be deployed in clinical practice.

## Conclusion and perspective

A critical need remains for developing a highly sensitive, inexpensive, and rapid diagnostic to identify neonates with cCMV. Targeted HCMV screening in neonates with abnormal hearing screen tests has facilitated the prompt diagnosis of cCMV, enabling the timely initiation of antiviral treatment and focused observation in early intervention programs.^107,108^ However, the CHIMES Study^109^ found that this strategy failed to detect 43% of infants with cCMV who subsequently developed SNHL during childhood. Moreover, the outcome data for 90% of neonates with asymptomatic cCMV infections are restricted to limited observations over the first few years of life, hindering an accurate determination of potential long-term adverse neurodevelopmental complications, including SNHL. More extensive population-based studies and universal screening initiatives are required to correctly define the effects of cCMV and determine if any intervention would prevent or attenuate observed impairments once detected.

Over the last five years, significant advances in defining the pathophysiology of HCMV in the human host have occurred. Viral and host genetic factors that fine-tune the delicate balance between lytic, latent, and reactivation stages of infection have been described. Incorporating this knowledge into emerging diagnostics may allow more accurate quantification of viral load in different tissue matrices, provide pertinent clinical information to distinguish between the different stages of infection, enable the detection of viral reactivation, and rapidly identify emerging antiviral resistance. However, financial, institutional, and educational support are imperative to accelerate the development of innovative and novel diagnostic and therapeutic approaches to this very common and devastating viral infection.

## Data Availability

Data sharing not applicable to this article as no datasets were generated or analyzed for this submission.
